# Restoration of Asymmetric Dimethylarginine–Nitric Oxide Balance to Prevent the Development of Hypertension

**DOI:** 10.3390/ijms150711773

**Published:** 2014-07-02

**Authors:** You-Lin Tain, Li-Tung Huang

**Affiliations:** 1 Department of Pediatrics, Kaohsiung Chang Gung Memorial Hospital, Chang Gung University College of Medicine, Kaohsiung 833, Taiwan; E-Mail: tainyl@hotmail.com; 2 Center for Translational Research in Biomedical Sciences, Kaohsiung Chang Gung Memorial Hospital, Chang Gung University College of Medicine, Kaohsiung 833, Taiwan; 3 Department of Traditional Chinese Medicine, Chang Gung University, Taoyuan 333, Taiwan

**Keywords:** asymmetric dimethylarginine, nitric oxide, hypertension, prehypertension, programmed hypertension

## Abstract

Despite the use of extensive antihypertensive therapy in patients with hypertension, little attention has been paid to early identification and intervention of individuals at risk for developing hypertension. The imbalance between nitric oxide (NO) and reactive oxygen species (ROS) resulting in oxidative stress has been implicated in the pathophysiology of hypertension. NO deficiency can precede the development of hypertension. Asymmetric dimethylarginine (ADMA) can inhibit nitric oxide synthase (NOS) and regulate local NO/ROS balance. Emerging evidence supports the hypothesis that ADMA-induced NO–ROS imbalance is involved in the development and progression of hypertension. Thus, this review summarizes recent experimental approaches to restore ADMA–NO balance in order to prevent the development of hypertension. Since hypertension might originate in early life, we also discuss the putative role of the ADMA–NO pathway in programmed hypertension. Better understanding of manipulations of the ADMA–NO pathway prior to hypertension in favor of NO will pave the way for the development of more effective medicine for the treatment prehypertension and programmed hypertension. However, more studies are needed to confirm the clinical benefit of these interventions.

## 1. Introduction

Hypertension is a highly prevalent disease globally that might originate during early life. Oxidative stress is a persistent oxidative shift that characterizes a pathological state, mainly caused by the imbalance between reactive oxygen species (ROS) and nitric oxide (NO). Oxidative stress and NO deficiency have been implicated in the pathophysiology of hypertension [1,2,3]. NO deficiency can precede the development of hypertension [4,5]. In addition, emerging evidence supports that NO–ROS imbalance is important for programmed hypertension [6,7,8].

NO deficiency can be caused by decreased nitric oxide synthase (NOS) expression/activity, decreased l-arginine availability (the substrate for NOS), inactivation due to oxidative stress, and inhibition by asymmetric dimethylarginine (ADMA, an endogenous NOS inhibitor). Among the reasons for NO–ROS imbalance, increasing attention has been centered on ADMA [9]. ADMA can reduce the synthesis of NO; however, it induces superoxide production by uncoupling NOS. Thus, cellular ADMA concentrations tightly regulate the local NO–ROS balance [9,10]. The kidney is an important long-term regulator of blood pressure and has been identified as a key player in programmed hypertension [11]. A better understanding of the role of the ADMA–NO pathway, specifically in the kidney, in the development of hypertension will allow us to develop ideal therapeutic strategies for patients with prehypertension.

This review aims to summarize evidence linking NO–ROS imbalance to the development of hypertension, with an emphasis on various manipulations of the ADMA–NO pathway prior to hypertension in favor of NO as a therapeutic approach for prehypertension and programmed hypertension.

## 2. Asymmetric Dimethylarginine (ADMA): A Link between Nitric Oxide (NO) and Reactive Oxygen Species (ROS) in the Development of Hypertension

Several groups, including our own, have found that ADMA is involved in the development and progression of hypertension [8,9,10,11,12,13,14]. Free ADMA levels are controlled by the counterbalancing type I protein arginine methyltransferase (PRMT) and dimethylarginine dimethylaminohydrolase (DDAH) pathways. PRMT-1 is the major type I PRMT enzyme responsible for ADMA synthesis, whereas DDAH-1 and -2 are responsible for ADMA breakdown [9,13]. ADMA can also be transported to other organs by cationic amino acid transporter (CAT) or renally excreted. ROS has been shown to increase PRMT-1 and inhibit DDAH activity leading to an increase in ADMA [15,16,17]. However, the NOS isoenzymes become uncoupled in the presence of high ADMA levels, further contributing to the oxidative stress burden. That is, ADMA can cause superoxide production but inhibit NO synthesis. Thereby, ADMA is considered as a major player leading to NO–ROS imbalance. Even though elevated ADMA levels were reported in human hypertension and diverse animal models of hypertension [9,10,12,13], little attention has been paid to elucidate whether ADMA induces NO–ROS imbalance and consequently programmed hypertension. Given that intracellular ADMA levels are mainly regulated by PRMT and DDAH, specific PRMT inhibitors or DDAH agonists might become novel therapeutic strategies to restore ADMA–NO and prevent hypertension.

## 3. The Spontaneously Hypertensive Rat (SHR) as a Model for Developmental Programming of Hypertension

The spontaneously hypertensive rat (SHR) is a widely employed experimental model of hypertension. The SHR model is characterized by a rise in blood pressure (BP) starting from 5 to 6 weeks of age, a steep increase between 6 and 24 weeks of age, and a progressive development of many features of hypertensive end-organ damage [18]. Interestingly, the SHR is more resistant to kidney damage than the salt-sensitive hypertensive rat [19].

Early renal NO deficiency, a predecessor of hypertension, is a characteristic of the SHR [4]. Several factors causing NO deficiency develop early in the kidney even before the onset of spontaneous hypertension, including increased ADMA, decreased l-arginine to ADMA ratio (AAR), increased oxidative stress, and increased protein inhibitor of neuronal nitric oxide synthase (PIN) expression [5]. In the hypertensive SHR, our data revealed that decreased renal cortical neuronal nitric oxide synthase-α (nNOS-α) protein level, increased renal PIN expression, increased plasma ADMA, decreased plasma AAR, and increased oxidative stress are the major causes for NO deficiency [5].

## 4. ADMA–NO Pathway: A Therapeutic Target for the Development of Hypertension in the SHR

**Figure 1:**
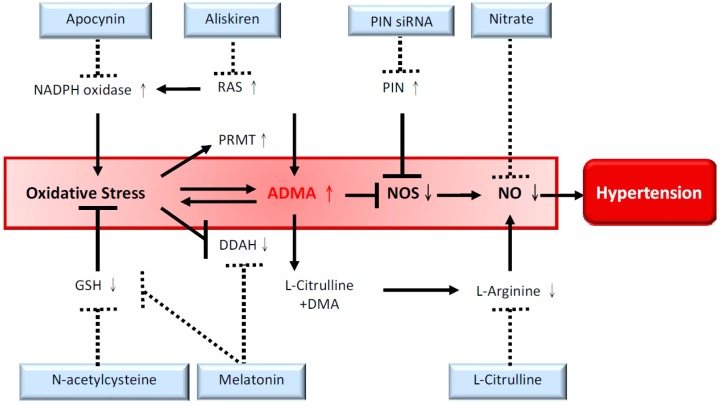
Overview of various therapeutic approaches to reduce ADMA and restore NO bioavailability to prevent the development of hypertension in spontaneously hypertensive rats. The solid lines represent underlying mechanisms contributing to hypertension, and the interrupted lines denote protective effects of different approaches. ADMA, asymmetric dimethylarginine; DDAH, dimethylarginine dimethylaminohydrolase; NADPH, nicotinamide adenine dinucleotide phosphate; DMA, dimethylamine; GSH, glutathione; NOS, nitric oxide synthase; PIN siRNA, silencing RNA targeting protein inhibitor of neuronal nitric oxide synthase; PRMT, protein arginine methyltransferases; RAS, rennin–angiotensin system.

Given the complex interaction between oxidative stress and ADMA, and, that a specific ADMA-lowering agent remains unavailable [9], our recent reports suggested several lines of evidence to support that restoration of ADMA–NO balance can prevent the development of hypertension in the SHR (Figure 1).

First, melatonin, an indoleamine produced from the pineal gland, is formed predominantly during the night. A growing body of evidence indicates that melatonin may regulate BP in experimental and human hypertension [20,21]. Our study showed that melatonin blocks the development of hypertension in SHRs through reduction of plasma ADMA, restoration of plasma AAR, preservation of renal l-arginine availability, and attenuation of oxidative stress [22]. Given that melatonin prevents the increase of ADMA and oxidative stress concurrently, we further determined if ROS-induced ADMA accumulation by regulation of DDAH can be prevented by melatonin. Our data suggests that the expression and activity of DDAH were suppressed *in vitro* by superoxide and hydrogen peroxide in a time-dependent manner, whereas melatonin could block H_2_O_2_-induced down-regulation of DDAH-2 and decrease DDAH activity, thereby preventing increases in ADMA [17]. Our findings reveal a mechanistic basis of DDAH down-regulation by ROS and suggest that melatonin shifting disturbed the NO–ROS balance in the prehypertension stage toward augmentation of NO, leading to lower blood pressure in young SHRs [22].

Second, observations have been made which show that apocynin blocks nicotinamide adenine dinucleotide phosphate (NADPH) oxidase to attenuate hypertension but has little effect on the ADMA–NO pathway in young SHRs [23]. Excessive ROS has emerged as a central common pathway, resulting in decreased NO bioavailability and decreased antioxidant capacity in the kidney, leading to hypertension. Two major sources of excessive ROS in hypertension are NADPH oxidase and uncoupling NOS. NADPH oxidase-derived ROS and ADMA are both increased in hypertension [1,3]. Apocynin, an NADPH oxidase inhibitor, can block NADPH oxidase assembly by interfering with the binding of p47phox to NOX. We found that apocynin prevented p47phox translocation in SHR kidneys, but not the increase of superoxide and H_2_O_2_ [23]. Additionally, apocynin did not protect SHRs against increased ADMA and only had a mild antihypertensive effect on SHRs. Our data suggest that simultaneous reduction of ROS and preservation of NO might be a better approach to restore ROS–NO balance to prevent the development of hypertension.

Third, there are studies showing that silencing RNA (siRNA) targeting PIN restores NO bioavailability and attenuates hypertension in SHRs [24]. The PIN was reported to inhibit neuronal NOS (nNOS) activity through disruption of nNOS dimerization [25]. PIN has also been shown to inhibit other NOS isoforms [26]. We found renal PIN expression was increased in pre-hypertensive and hypertensive stages in SHRs. Inhibition of PIN expression by siRNA attenuates the development of hypertension in SHRs at 12 weeks of age, which is related to decreased oxidative stress [24]. These findings support the hypothesis of restoring nNOS–NO to restore NO bioavailability and prevent the transition from pre-hypertension to hypertension.

Fourth, glutathione (GSH) is the major intracellular antioxidant. The GSH system is impaired in young SHR kidneys prior to the development of hypertension [27]. *N*-Acetylcysteine (NAC), an antioxidant, can facilitate GSH synthesis. NAC treatment attenuates the development of hypertension in young SHRs, which is correlated with a reduction in plasma ADMA levels, a decrease in superoxide production, an increase in DDAH activity, and an increase in GSH to oxidized GSH ratio in the SHR kidney [28]. These observations indicate that NAC can restore the NO–ROS balance, thus preventing the development of hypertension. Our findings also highlight the impact of GSH on programmed hypertension by regulating the DDAH–ADMA pathway. Consistent with other reports, antioxidant treatments initiated at the prenatal stage can prevent BP programming in SHRs [29,30].

Last, l-arginine has been shown to reduce systemic BP in some forms of experimental hypertension [31]. l-Citrulline supplementation enhances NO production more than l-arginine itself because it bypasses splanchnic extraction and it is not a substrate for arginase [32]. In line with a previous study [4], our recent report demonstrated that l-citrulline supplementation prevents the transition from prehypertension to hypertension in young SHRs [33]. This therapeutic effect of l-citrulline is associated with the bioactivation of the NO pathway, including reduced ADMA, increased AAR, augmented nNOS-α protein abundance, and increased NO production in SHR kidneys. On the other hand, nitrate and nitrite are the main substrates to produce NO via the NOS-independent pathway. Our study showed that dietary supplementation of nitrate, in amounts resembling a rich intake of vegetables in humans, can prevent the development of hypertension in young SHRs [33]. Our data are in agreement with a previous study showing that inorganic nitrate can lower BP in a hypertensive model [34]. Thus, our report suggests both NOS-dependent and independent approaches can restore NO bioavailability and reduce BP in SHR kidneys [33].

The data described above suggest that targeting the ADMA–NO pathway prior to hypertension in favor of NO could be a therapeutic approach to prevent the transition from prehypertension to the hypertensive stage in SHRs.

## 5. ADMA–NO Pathway: A Therapeutic Target for Programmed Hypertension

It is well established that early-life environmental insults during critical periods of development can elicit impaired nephrogenesis, morphological changes, and adaptive physiological responses, leading to hypertension in adult life [35]. Our recent studies indicated that ADMA-induced NO–ROS imbalance is involved in the development of hypertension in different developmental models, including maternal caloric restriction, maternal diabetes, and prenatal dexamethasone exposure [8,14,36]. However, manipulation of the ADMA-NO pathway by l-citrulline supplementation can prevent these conditions [8,14,36].

In addition to oxidative stress, other proposed mechanisms involved in developmental programming of hypertension include alterations of the renin-angiotensin system (RAS). The RAS plays a fundamental role in the regulation of BP and kidney development. Studies showing that the blockade of RAS by angiotensin-converting enzyme (ACE) inhibitor captopril or angiotensin II type 1 receptor blocker (ARB) losartan between 2 and 4 weeks of age offsets the effects of nutritional programming on BP and were supportive of the role of RAS, linking maternal malnutrition to adulthood hypertension [37,38]. We used to treat young SHRs with the direct renin inhibitor, aliskiren. We found that aliskiren can reduce ADMA and prevent the development of hypertension in young SHRs [39]. Treatment of male offspring of rats subjected to maternal caloric restriction with aliskiren or losartan between the ages of 2 and 4 weeks postnatally prevents the development of hypertension at 12 weeks of age. Interestingly, prevention of the elevation of BP with aliskiren therapy in caloric restriction (CR) offspring is related to restoration of ADMA–NO balance, too. We found that aliskiren therapy decreased ADMA levels but increased AAR in the plasma, which was consistent with our recent findings showing that ADMA contributed to programmed hypertension and that aliskiren had an ADMA-lowering effect [40]. Unlike aliskiren, losartan decreases BP but not ADMA. Compared with aliskiren, losartan’s BP-lowering effect is similar. However, losartan had no effects on ADMA and NO bioavailability. Our data demonstrate that there is a critical window in the early postnatal period during which the adult BP can be modified by blockade of the RAS and which is, at least in part, dependent on the ADMA–NO pathway.

## 6. Are We Ready to Apply ADMA–NO Pathway into Clinical Practice?

Currently, the diagnosis of hypertension throughout the world is accomplished by clinical BP assessment, many by a few daytime measurements. Patients with prehypertension who are at risk for other BP abnormalities (e.g., nocturnal hypertension, increased BP load, and non-dipping nocturnal BP) will be missed if they are not assessed using 24 h ambulatory blood pressure monitoring (ABPM). Basic and clinical research performed in the last 20 years has implicated ADMA as a novel risk factor, diagnostic marker, and therapeutic target in cardiovascular disease, including hypertension [41]. Nevertheless, very little attention has been paid to target ADMA–NO in prehypertension and programmed hypertension clinically, largely because of the following blocks: (1) a specific ADMA-lowering agent remains unavailable; (2) measurements of ADMA, NO, and 24 h ABPM in patients are not yet performed on a routine basis; (3) the use of antihypertensive drugs for prehypertension remains debatable; and (4) indices that represent imbalanced ADMA–NO pathway from experimental models are required for clinical translation to non-invasive cardiovascular assessments. While a few small-scale studies indicate that ADMA is related to abnormal ABPM profile [42,43,44] and index of arterial stiffness [45,46] in patients with prehypertension, more large, prospective, multicenter collaborations are required to conduct meaningful clinical research studies to explore the impact of the ADMA–NO pathway in clinical practice.

## 7. Conclusions

Patients with prehypertension have an increased risk of full-blown hypertension, target organ damage, and cardiovascular morbidity and mortality [47]. Arguments against the use of antihypertensive drugs for prehypertension include a lack of evidence of efficacy and cost-effectiveness. Thus, reducing the future burden of hypertension will require early detection of individuals that are at risk for prehypertension and early treatment to delay the progression to full-blown hypertension.

In conclusion, this review provides an overview of experimental approaches to restore ADMA–NO imbalance to prevent hypertension: (1) it discusses how ADMA links NO–ROS balance to programmed hypertension; (2) it presents a series of therapeutic approaches to prevent hypertension in SHRs, including melatonin, apocynin, siRNA targeting PIN, *N*-acetylcysteine, l-citrulline, and sodium nitrate; (3) it discusses how early blockade of RAS can prevent programmed hypertension and its relationship to the ADMA–NO pathway; and (4) it indicates problems that must be addressed before restoration of ADMA–NO can be translated into clinical practice.

## Abbreviations

AAR: l-arginine to ADMA ratio
ACE: angiotensin-converting enzyme
ADMA: asymmetric dimethylarginine
ARB: angiotensin II type 1 receptor blocker
CAT: cationic amino acid transporter
DDAH: dimethylarginine dimethylaminohydrolase
GSH: glutathione;
NAC: *N*-acetylcysteine
nNOS: neuronal nitric oxide synthase
NO: nitric oxide
NOS: nitric oxide synthase
PIN: protein inhibitor of neuronal nitric oxide synthase
PRMT: protein arginine methyltransferase
RAS: rennin–angiotensin system
ROS: reactive oxygen species
SDMA: symmetric dimethylarginine
SHR: spontaneously hypertensive rat
BP: blood pressure
